# Development of a 7-DOF robotic arm control system for autonomous grasping of user-specified general objects

**DOI:** 10.3389/frobt.2026.1955232

**Published:** 2026-10-21

**Authors:** Shuting Bai, Yinlai Jiang, Hiroshi Yokoi, Shunta Togo

**Affiliations:** 1 Department of Mechanical and Intelligent Systems Engineering, The University of Electro-Communications, Tokyo, Japan; 2 Center for Neuroscience and Biomedical Engineering, The University of Electro-Communications, Tokyo, Japan

**Keywords:** 6-DOF grasp pose estimation, 7-DOF robotic arm, autonomous grasping, inverse kinematics, mixture of experts, multi-task learning, point cloud, semantic segmentation

## Abstract

Robotic manipulators used in assistive robots need the ability to autonomously grasp various objects in unstructured environments and allow users to specify target objects in cluttered environments. This study developed a control system for autonomous grasping of user-specified general objects on a coupled tendon-driven 7-DOF robotic arm. A geometry-based inverse kinematics method was introduced to generate grasping motions for the 7-DOF robotic arm, and an end-to-end multi-task model based on the proposed 3D Mixture-of-Experts (MoE) UNet was developed to simultaneously perform 6-DOF grasp pose estimation and semantic segmentation using point cloud data as input. Evaluation on the GraspNet-1Billion dataset showed that the proposed 3D MoE UNet backbone generally improved both tasks over the 3D UNet baseline, achieving gains of up to 5.24 AP points for grasp pose estimation and up to 3.42 mIoU points for semantic segmentation, while requiring only a limited number of additional active parameters during inference. In real-world grasping experiments involving 10 objects in cluttered scenes, the system achieved a 100% pick-and-place success rate for each of the three seen objects and an average success rate of 52.9% for the 7 unseen objects. During these experiments, the proposed model operated in real time at an average of 20 FPS with limited memory usage. These results demonstrate the effectiveness of the proposed system for autonomous grasping in cluttered real-world environments and its potential for application to standalone assistive robots.

## 1 Introduction

In recent years, rapid advances in machine learning have significantly enhanced the autonomy and functionality of industrial and service robots. In particular, the research, development, and practical application of collaborative robots (cobots) (Peshkin and Colgate, 1999) which operate alongside humans in environments such as factories and warehouses, as well as assistive robots (Feil-Seifer and Mataric, 2005; Van der Loos and Reinkensmeyer, 2008; Chen et al., 2013) that assist people with disabilities and the elderly with their daily lives at home have progressed rapidly. In these robots, robotic manipulators which are responsible for grasping and manipulating objects play a crucial role in performing a wide variety of tasks in human living and working environments.

Conventional industrial robotic manipulators are widely used as substitutes for human workers in automotive manufacturing and electronics production, contributing to improved productivity and consistent quality. In terms of control methods, since these systems are specialized for transporting and assembling specific objects in structured environments, they primarily rely on precisely tracking predefined trajectories. In contrast, robotic manipulators for cobots and assistive robots are required to safely operate in close proximity to humans while performing a wide variety of tasks in complex environments, including object grasping and transportation as well as tool manipulation. To accomplish these tasks, the robot must perceive the surrounding environment and autonomously determine appropriate grasping actions while accommodating uncertainties in the shape and pose of target objects. In particular, since assistive robots are expected to manipulate a wide variety of household objects in unstructured domestic environments, there is a need for manipulators with degrees of freedom comparable to those of the human upper limb to enable obstacle avoidance and flexible motion generation. Therefore, the development of advanced perception and control algorithms based on sensory information, particularly visual information, remains a critical research challenge.

Over recent years, numerous deep learning-based grasp pose estimation methods using RGB-D images or point cloud data have been proposed. Various approaches have been studied, including methods that combine the generation and evaluation of candidate grasp poses (ten Pas et al., 2017; Liang et al., 2018) and end-to-end methods that directly regress the grasp pose (Redmon and Angelova, 2014; Morrison et al., 2018; Qin et al., 2020). By leveraging point cloud feature extraction based on PointNet-based networks (Qi et al., 2016; 2017) and training with large-scale datasets, these methods have achieved high generalization performance for previously unseen general objects (Mahler et al., 2017; Fang et al., 2020; Wang et al., 2021). However, although many studies have focused on improving the accuracy and efficiency of grasp pose estimation, few have comprehensively addressed the challenges associated with practical deployment, such as the application to manipulators with redundant degrees of freedom and user-specified target object selection in cluttered environments containing multiple objects.

Accordingly, the objective of this study is to develop a practical robotic manipulator control system for assistive robots that seamlessly integrates grasp pose estimation for general objects, user-specified target object selection, and autonomous reach-to-grasp movement control. To this end, a coupled tendon-driven 7-DOF robotic arm developed in previous studies (Li et al., 2021; Nakamura et al., 2022) is adopted as the control target, and a geometry-based inverse kinematics method for 7-DOF manipulators is employed. Furthermore, this study proposes an end-to-end multi-task neural network model that simultaneously performs 6-DOF grasp pose estimation and semantic segmentation from point cloud data for multiple general objects in cluttered environments. The proposed model is evaluated on a publicly available benchmark dataset (Fang et al., 2020), demonstrating superior performance compared with previous methods and the ability to operate in real time with relatively limited computational resources. Finally, the proposed method is integrated into the physical coupled tendon-driven 7-DOF robotic arm, and reach-to-grasp experiments are conducted on 10 different general objects to validate the effectiveness of the proposed control system.

The main contributions of this study are as follows.

1. We proposed a 3D Mixture-of-Experts (MoE) UNet based on MinkUNet14 (Choy et al., 2019; Ronneberger et al., 2015) as the backbone of the multi-task model, which generally improved the performance of both 6-DOF grasp pose estimation and semantic segmentation compared with the other evaluated backbones.
2. We developed a multi-task framework that jointly performs 6-DOF grasp pose estimation and semantic segmentation within a single model, reducing the computational resource requirements during inference compared with using separate models for the two tasks.
3. We developed and experimentally validated an integrated robotic grasping system by combining the proposed method with a previously developed coupled tendon-driven 7-DOF robotic arm and a geometry-based inverse kinematics method.

## 2 Related works

Reach-to-grasp movement and manipulation are fundamental capabilities of robotic manipulators and have been extensively studied since the 1970s (Shimoga, 1996). Estimating a grasp pose that allows for accurate and stable grasping of an object is a critical challenge in the automatic control of reach-to-grasp movements. With recent advances in sensor technology and computer vision, research on machine learning-based grasp pose estimation using visual information has become increasingly active. Depending on the degrees of freedom of the grasp representation, vision-based grasp pose estimation tasks can be broadly classified into two types: grasp rectangle detection task, which represents a grasp as a rectangle in the image plane, and 6-DOF grasp pose estimation task, which handles grasp poses in three-dimensional (3D) space.

For the grasp rectangle detection task, an RGB-D image is used as the input, and the predicted grasp is represented as a rectangle in the image plane, parameterized by the grasp center, in-plane rotation angle, and gripper width. As a representative early work, Lenz et al. (2013) proposed a method that generates a large number of candidate grasp rectangles using a sliding-window approach and classifies their graspability with a convolutional neural network (CNN). Redmon and Angelova (2014) formulated grasp rectangle detection as a regression problem and proposed a CNN-based method that directly predicts the parameters of the grasp rectangle at the grid level from the entire input image, enabling real-time graspable rectangle estimation. Furthermore, Morrison et al. (2018) proposed the Generative Grasping Convolutional Neural Network (GG-CNN), which employs fully convolutional network architecture to predict the parameters of grasp rectangles on a pixel-by-pixel basis, achieving both real-time performance and high accuracy. To improve the generalization capability of grasp detection models, Mahler et al. (2017) generated synthetic depth images from 1,500 CAD models and automatically annotated grasp candidates by computing a grasp quality metric based on contact mechanics, thereby constructing a large-scale dataset. They further proposed the Grasp Quality Convolutional Neural Network (GQ-CNN) to evaluate the robustness of grasp candidates. Experimental results on the constructed dataset demonstrated that the proposed method achieves fast grasp planning while maintaining strong generalization performance on previously unseen objects. Although these methods are computationally efficient and exhibit a certain degree of generalization to previously unseen objects, they cannot fully exploit the three-dimensional geometric characteristics of the target object and its surrounding environment because they assume top-down grasping in a planar setting. As a result, they are unable to represent side grasps for tall objects with a high center of mass and cannot handle scenarios in which the target object is partially occluded or located beneath other objects.

To overcome these limitations, extensive research has recently been conducted on 6-DOF grasp pose estimation in 3D space using RGB-D images or point clouds. As a representative early work, ten Pas et al. (2017) proposed a method that first randomly generates a large number of 6-DOF grasp pose candidates from point cloud data using a rule-based approach. Then, the local point cloud around each grasp candidate is projected onto a 2D image, and its grasp quality is evaluated using a CNN that takes the projected image as input. Although this method can estimate grasp poses for arbitrary objects, it requires the generation of a large number of grasp candidates, resulting in a high computational cost and limiting its real-time performance. Moreover, the success rate of grasp execution is highly dependent on the quality of the randomly generated grasp candidates. Building upon the method proposed by ten Pas et al. (2017), Liang et al. (2018) directly processed the local point cloud around each grasp pose candidate using a PointNet-based (Qi et al., 2016) network, thereby enhancing the accuracy of grasp quality evaluation. By operating directly on point clouds rather than projected images, the method preserves the three-dimensional geometric information of the scene and achieves better performance when handling objects with complex shapes. Furthermore, Mousavian et al. (2019) formulated grasp generation as a distribution estimation problem and proposed a method that learns the distribution of grasp poses from an object’s point cloud using a Variational Autoencoder (VAE) (Kingma and Welling, 2013), enabling the generation of multiple grasp poses for a single object. Because grasp pose candidates are generated according to the learned probability distribution rather than through random sampling, the computational cost is reduced.

However, as with previous methods, the generated grasp poses still require an additional grasp quality evaluation. Qin et al. (2020) proposed a single-shot method for regressing 6-DOF grasp poses for multiple objects in cluttered environments from partial point clouds acquired by a single-view depth sensor. Their method employs a PointNet++-based (Qi et al., 2017) network to directly predict grasp poses in a single forward pass. Unlike conventional approaches that separate grasp pose generation from grasp quality evaluation, the proposed framework directly estimates grasp poses using a single network and therefore enables fast inference. Subsequently, Fang et al. (2020) constructed a large-scale dataset containing more than 1 billion annotated grasp poses from real-world cluttered scenes and proposed a benchmark for evaluating grasping performance on general objects in real environments. Furthermore, rather than directly regressing 6-DOF grasp poses in SE (3) space, they proposed a contact-based grasp representation that first estimates graspable contact points, surface normal directions, and in-plane rotation angles, from which the corresponding 6-DOF grasp poses are reconstructed. Building upon the method of Fang et al. (2020), Wang et al. (2021) introduced a new metric termed graspness, to quantify how suitable each point in a point cloud is for grasping. They incorporated an independent graspness prediction module into the grasp pose estimation network and sampled only the points with high graspness scores as grasp contact points. As a result, the method can efficiently generate high-quality grasp poses.

Overall, 6-DOF grasp pose estimation has evolved from two-stage approaches that separately generate and evaluate grasp pose candidates to end-to-end methods that directly regress grasp poses. Building upon these previous studies, in this work we propose a multi-task model that simultaneously performs 6-DOF grasping pose estimation and semantic segmentation for multiple general objects in cluttered environments using point cloud data as input. By leveraging the semantic segmentation results, the proposed model can identify the object associated with each estimated grasp pose and present this information to the user, thereby enabling user-specified target object selection in cluttered environments. Furthermore, the proposed point cloud feature extraction backbone not only achieves superior performance in the proposed multi-task model but also improves the performance of a single-task grasp pose estimation model, outperforming previous methods.

## 3 Materials and methods

### 3.1 Coupled tendon-driven 7-DOF robotic arm

In this study, a coupled tendon-driven 7-DOF robotic arm developed by Nakamura et al. (2022) is employed. This robotic arm is based on the design proposed by Li et al. (2021) and incorporates a return-routing tendon-driven mechanism into the shoulder module, thereby reducing the module length and achieving dimensions that more closely resemble those of the human upper limb. Because the wires are lightweight and elastic, they offer a high level of safety and are well-suited for application in assistive robots that collaborate with humans. As shown in Figure 1, the robotic arm consists of three coupled tendon-driven modules: a 3-DOF shoulder joint module, a 2-DOF elbow joint module, and a 2-DOF wrist joint module. All motor outputs T are coupled within the modules, and the output torque τ is calculated using Equation 1:

$$\left[\begin{array}{c} \tau_{1}\\ \tau_{2}\\ \tau_{3}\\ \tau_{4}\\ \tau_{5}\\ \tau_{6}\\ \tau_{7} \end{array}\right]=\left[\begin{array}{ccccccc} 1 & 2 & -1 & 0 & 0 & 0 & 0\\ -1 & 1 & 1 & 0 & 0 & 0 & 0\\ -1 & 0 & -1 & 0 & 0 & 0 & 0\\ 0 & 0 & 0 & 1 & 1 & 0 & 0\\ 0 & 0 & 0 & -1 & 1 & 0 & 0\\ 0 & 0 & 0 & 0 & 0 & 1 & 1\\ 0 & 0 & 0 & 0 & 0 & -1 & 1 \end{array}\right]\left[\begin{array}{c} T_{m1}\\ T_{m2}\\ T_{m3}\\ T_{m4}\\ T_{m5}\\ T_{m6}\\ T_{m7} \end{array}\right]$$ (1)

**FIGURE 1:**
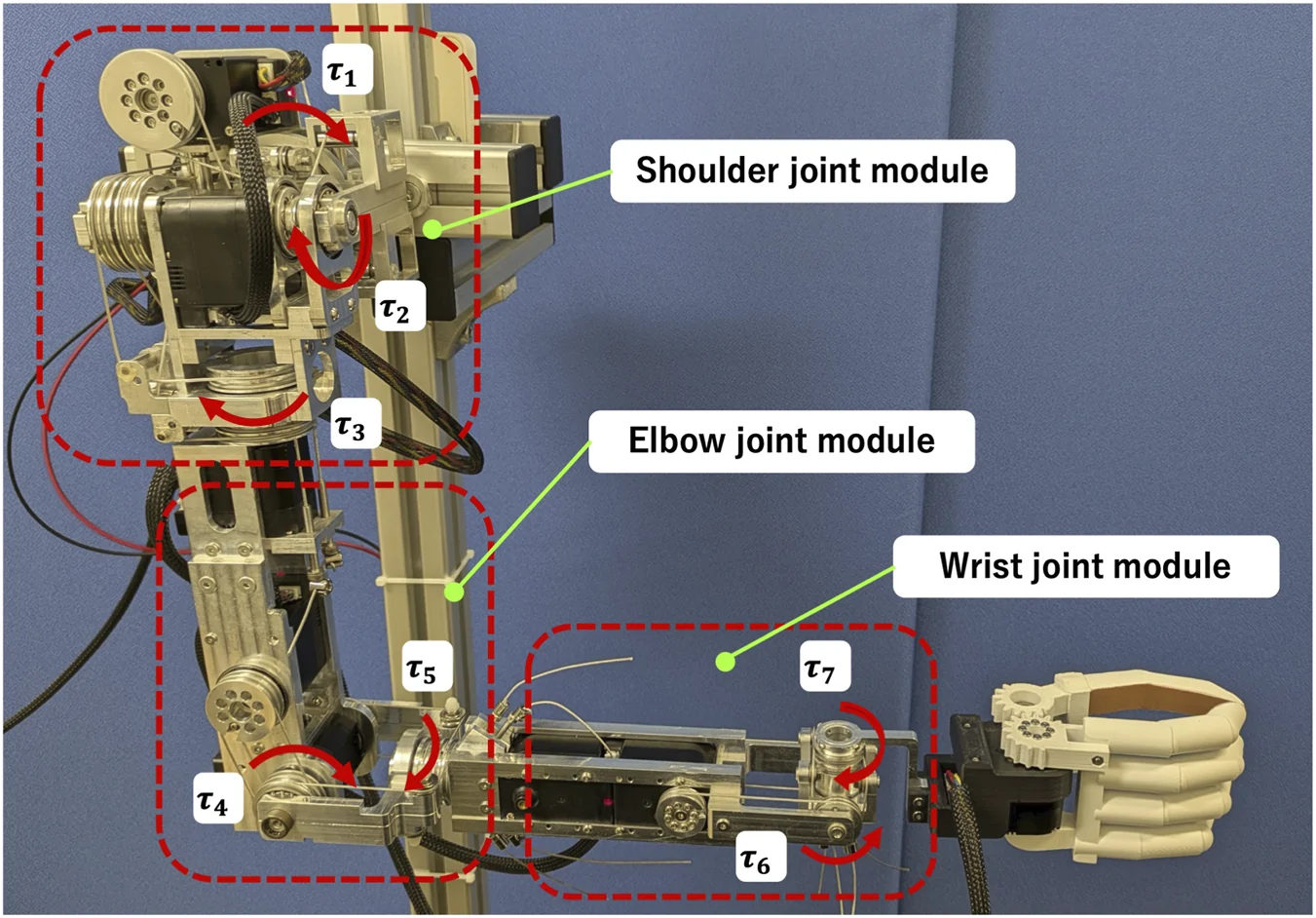
A 7-DOF robotic arm with coupled tendon-driven joints.

The motor-joint routing matrix used in this study was adopted directly from Li et al. (2021). The coefficients in the motor-joint routing matrix are determined by the pulley radius ratios and tendon routing. A coefficient of 1 corresponds to the case where the motor and joint pulleys have the same radius, while a coefficient of 2 is determined by the corresponding pulley radius ratio. The signs of the coefficients represent the relationship between the rotational directions of the motor and joint pulleys resulting from the tendon routing (Li et al., 2021).

The end-effector is a 1-DOF robotic gripper shaped like a human hand, capable of grasping objects by opening and closing the thumb and the remaining four fingers. The robotic arm weighs 2.2 kg and has a payload capacity of 1.5 kg. Since the objective of this study is to grasp general objects commonly found in household environments for use in assistive robots, a payload capacity of 1.5 kg is considered sufficient for the intended tasks.

### 3.2 Inverse kinematics for 7-DOF robotic arm

Since a 7-DOF robotic arm possesses kinematic redundancy, when the end-effector pose is fixed, the elbow joint can occupy infinitely many positions on a circle centered at the intersection between the line connecting the shoulder and wrist joints and the perpendicular dropped from the elbow joint to this line, as illustrated in Figure 2. Therefore, to solve the inverse kinematics using geometry-based methods, the elbow joint position must be uniquely determined. As shown in Figure 2, the plane defined by the shoulder, elbow, and wrist joints is referred to as the reference plane when it is perpendicular to the XY plane of the base coordinate system, and as the arm plane otherwise. The angle φ between the arm plane and the reference plane is defined as the arm angle.

**FIGURE 2:**
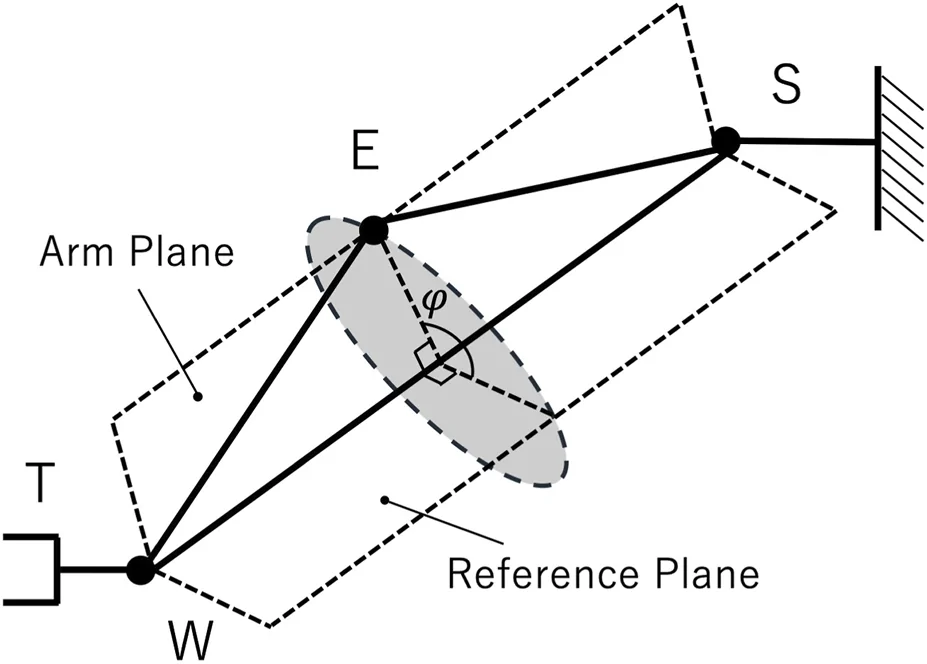
Kinematic redundancy of a 7-DOF robotic arm.

Similar to the human arm, the shoulder joint of the 7-DOF robotic arm used in this study adopts a roll–pitch–roll configuration, in which 2 of its 3 degrees of freedom are kinematically coupled with the arm angle. Therefore, even when the arm angle is specified, the shoulder joint angles cannot be uniquely determined. In contrast, among the 3 degrees of freedom consisting of pitch-yaw rotation of the wrist joint and roll rotation of the elbow joint, only the roll rotation of the elbow joint is coupled with the arm angle. Consequently, once the arm angle and the target end-effector pose are specified, the angle of the elbow joint’s roll rotation and the angles of all the remaining joints are uniquely determined. Based on the characteristics described above, this study employs the inverse kinematics method proposed by Dou et al. (2022), which utilizes a reverse order manipulator coordinate system. Using this approach, the problem of determining the joint angles required to move the end-effector to the target pose is reformulated as the problem of determining the joint angles required to move the base-link to its initial configuration while keeping the end-effector fixed at the target pose.

First, at the initial position of the robot arm, we define the reverse-order manipulator coordinate system according to the modified DH method, as shown in Figure 3. In this coordinate system, frame 0 corresponds to the end-effector link frame and frame 7 corresponds to the base-link frame.

**FIGURE 3:**
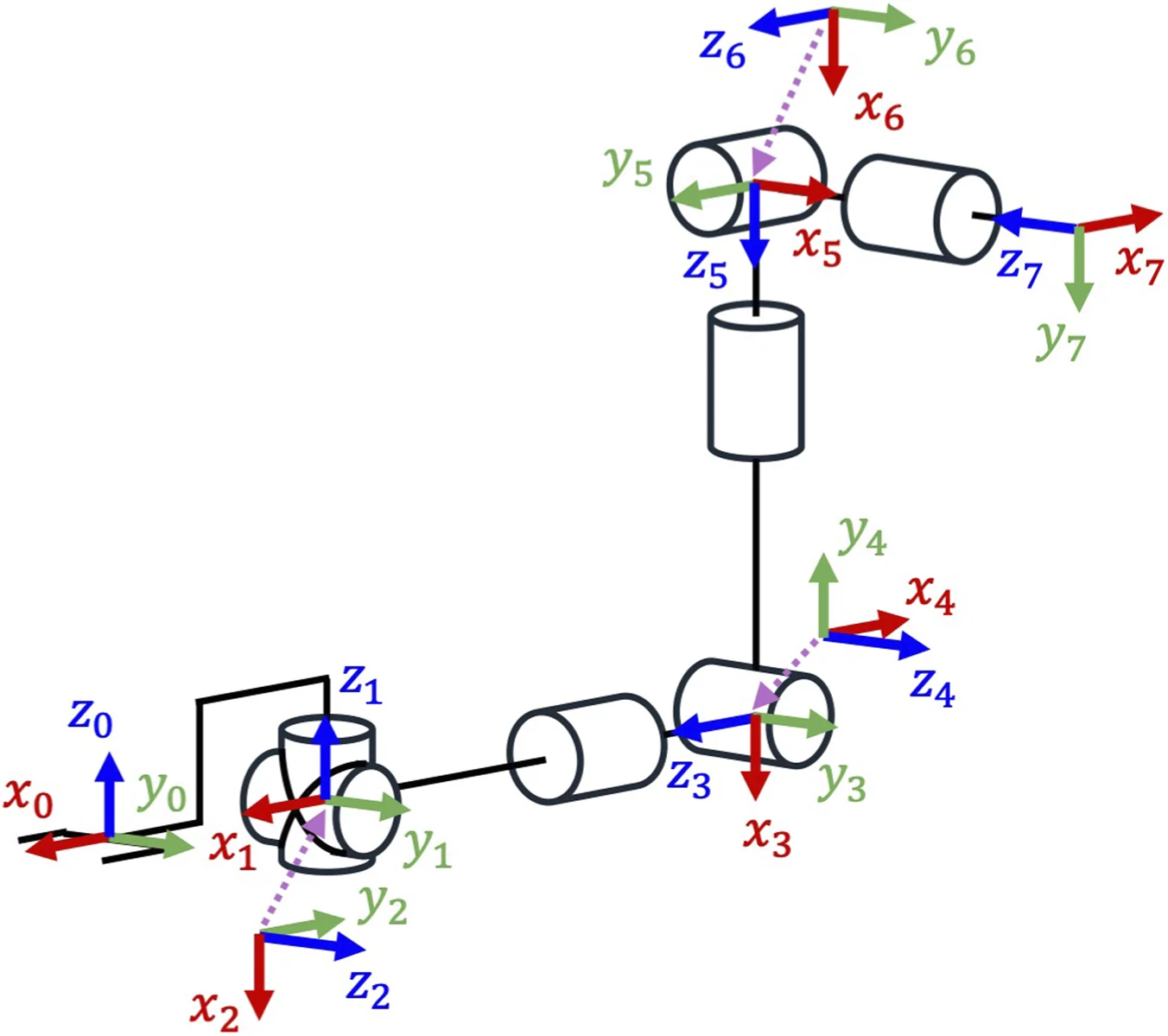
Reverse order manipulator coordinate system following the modified Denavit-Hartenberg (MDH) convention.

Given the target pose T07 of the end-effector expressed in the base-link frame, the target position P07 and orientation R07 of the end-effector, as well as the target position P70 and orientation R70 of the base-link expressed in the end-effector link frame, are uniquely determined.

Figure 4 illustrates the geometric relationship when the shoulder, elbow, and wrist joints lie on the reference plane. As shown in Figures 2, 4, the elbow joint angle $\theta_{4}$ depends solely on the end-effector position and can be calculated using the law of cosines as shown in Equation 2:

$$\theta_{4}=\arccos\left(\frac{{\left|L_{ws}\right|}^{2}-d_{we}^{2}-d_{es}^{2}}{2d_{we}d_{es}}\right)$$ (2)

where $L_{ws}$ denotes the wrist-shoulder vector, $d_{we}$ is the distance between the wrist and elbow joints, and $d_{es}$ is the distance between the elbow and shoulder joints.

**FIGURE 4:**
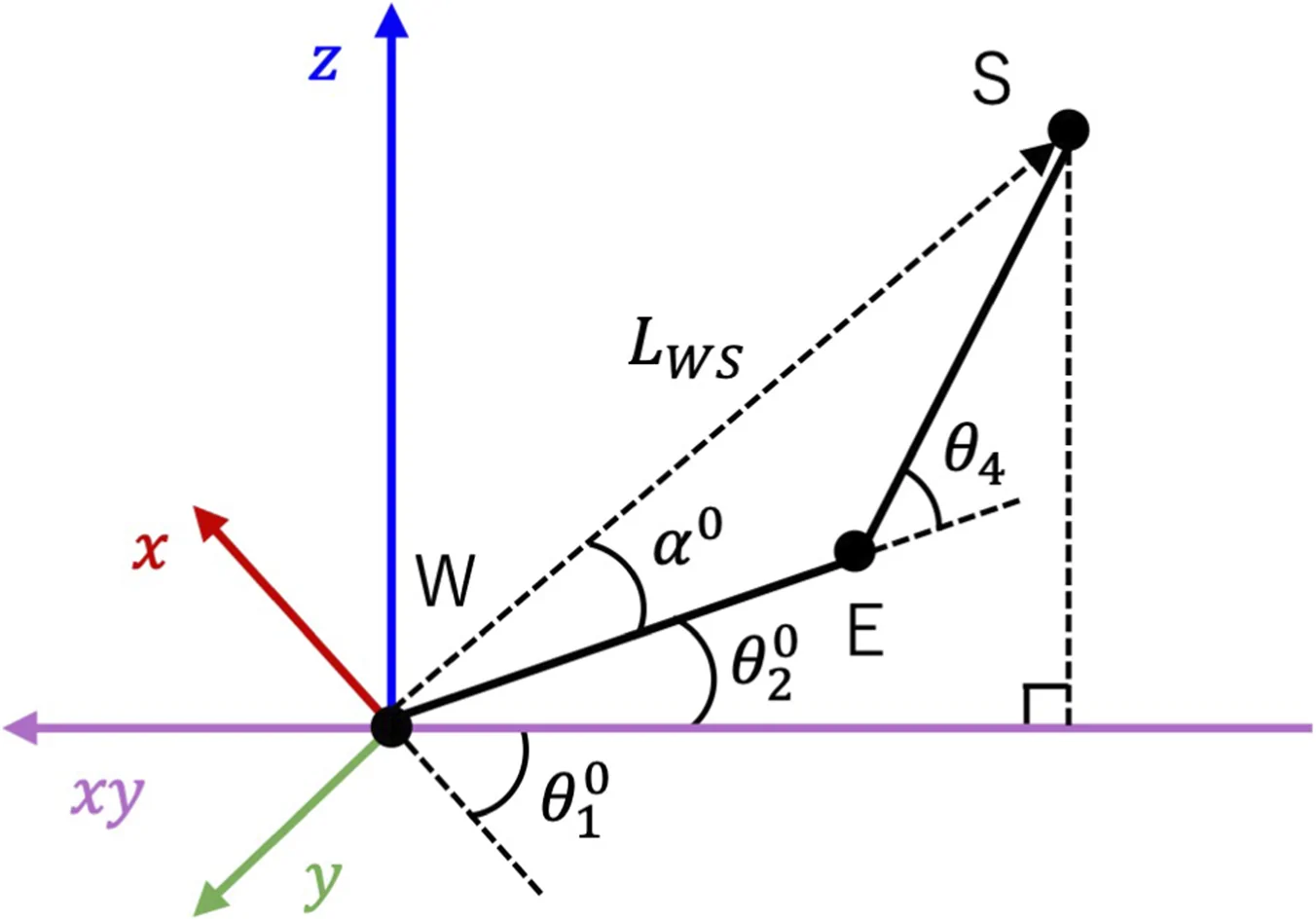
Geometric relationship among the shoulder, elbow, and wrist joints on the reference plane.

Next, the angles $\theta_{1}^{0}$ , $\theta_{2}^{0}$ , and $\theta_{3}^{0}$ of joints 1, 2, and 3, on the reference plane are computed. When the arm angle is 0, $\theta_{3}^{0}$ is uniquely determined to be 0. $\theta_{1}^{0}$ is the angle between the projection of $L_{ws}$ onto the xy plane and the x-axis, and is given by Equation 3:

$$\theta_{1}^{0}=\text{atan}2\;\left(\frac{L_{ws,y}}{L_{ws,x}}\right)$$ (3)

$\theta_{2}^{0}$ is the angle between the projection of $L_{ws}$ onto the XY plane and wrist-elbow vector, and is given by Equations 4–6:

$$\theta_{2}^{0}=\text{atan}2\;\left(L_{ws,z},-\text{sign }\left(L_{ws,x}\right)\sqrt{L_{ws,x}^{2}+L_{ws,y}^{2}}\right)-GC_{41}\alpha^{0}$$ (4)

$$GC_{41}=\left\{\begin{array}{cc} 1 & \left(\theta_{4}⩽\frac{\pi }{2}\right)\\ -1 & \left(\theta_{4}>\frac{\pi }{2}\right) \end{array}\right.$$ (5)

$$\alpha^{0}=\arccos\left(\frac{{\left|L_{ws}\right|}^{2}+d_{we}^{2}-d_{es}^{2}}{2\left|L_{ws}\right|d_{we}}\right)$$ (6)

Furthermore, the rotation matrix Rr30 which represents the coordinate transformation from frame 0 to frame 3, can be computed using Rodrigues’ rotation formula as shown in Equations 7, 8:

Rr30=R30φ0R0 (7)

Rφ0=I3+sin⁡φuws+1−cos⁡φuws2 (8)

where φ denotes the arm angle, and $\left[u_{ws}\right]$ is the skew-symmetric matrix corresponding to the unit vector of $L_{ws}$ . The rotation matrix R030 represents the coordinate transformation from frame 0 to frame 3 on the reference plane, and is obtained by substituting the previously calculated joint angles $\theta_{1}^{0}$ , $\theta_{2}^{0}$ , and $\theta_{3}^{0}$ into the rotation matrix R30 which is derived using the modified DH convention. Since both R30 and Rr30 represent the coordinate transformation from frame 0 to frame 3, their corresponding matrix elements are equal. By equating the corresponding elements of the two matrices, the angles $\theta_{1}$ , $\theta_{2}$ , and $\theta_{3}$ of joints 1, 2, and 3 are obtained as shown in Equations 9–11:

θ1=atan2 R23r30R13r30 (9)

θ2=arcsin−R33r30 (10)

θ3=atan2 R32r30−R31r30 (11)

where Rijr30 denotes the element in the i -th row and j -th column of Rr30 .

Then, the rotation matrix Rr74 from frame 4 to frame 7 is computed using the previously obtained Rr30 as shown in Equation 12:

Rr74=R⊤43Rr⊤30R70 (12)

Following the same procedure described above, the angles $\theta_{5}$ , $\theta_{6}$ , and $\theta_{7}$ of joints 5, 6, and 7 are obtained by equating the corresponding elements of the rotation matrices R74 which is derived using the modified DH convention and Rr74 as shown in Equations 13–15:

θ5=atan2 −R13r74R33r74 (13)

θ6=arcsin−R23r74 (14)

θ7=atan2 R21r74R22r74 (15)

where Rijr74 denotes the element in the i -th row and j -th column of Rr74 .

As described above, once the Arm Angle φ and the target end-effector pose T07 expressed in the base-link frame are specified, all joint angles of the 7-DOF robotic arm can be determined. In the implementation of the inverse kinematics solver, the search range of the arm angle is set from −0.26 rad to 0.78 rad . An exhaustive search is then performed by varying the arm angle in increments of 0.002 rad , and the arm angle that minimizes the sum of the absolute values of all computed joint angles is selected as the optimal solution.

To evaluate the reachable pose workspace of the robotic arm introduced in Section 3.1 using the inverse kinematics method described above, a simulation-based workspace analysis was conducted. The end-effector position was uniformly sampled within a three-dimensional region ranging from −0.70 to 0.70 m along the x , y , and z axes in the base-link frame, with a sampling interval of 0.04 m . For each sampled position, 50 approaching directions were uniformly distributed over a sphere using Fibonacci sphere sampling algorithm (González, 2009). For each approaching direction, the in-plane rotation angle around the end-effector approaching axis was varied from −90° to 90° at intervals of 30° , resulting in 350 candidate end-effector orientations at each position. Inverse kinematics was then computed for all sampled poses, with the range of each joint angle constrained according to the hardware structure of the robotic arm, and poses for which valid joint angles were obtained were regarded as reachable poses. The pose reachability ratio at each sampled position was then calculated as the ratio of reachable poses to all sampled poses at that position. During the above workspace analysis, the inverse kinematics computation time averaged approximately 1.3 ms per end-effector pose in our computational environment. The resulting reachable pose workspace and its cross-sectional distributions are shown in Supplementary Figure S1.

### 3.3 6-DOF grasp pose estimation and semantic segmentation

In practical deployment of assistive robots in real-world environments, the control system is required to estimate grasp poses for multiple objects in cluttered scenes, allowing the user to select the desired target object for grasping. To achieve this functionality, semantic segmentation needs to be performed on the scene to identify the object corresponding to each estimated grasp pose. However, introducing a separate semantic segmentation model in addition to the grasp pose estimation model substantially increases the computational resources required for inference. Since assistive robots are generally expected to operate as a standalone system, all perception and control processes are typically performed on a compact on-board edge computer, where the available computational resources are limited.

Motivated by the aforementioned challenges, this study proposes an end-to-end multi-task neural network model that simultaneously performs 6-DOF grasp pose estimation and semantic segmentation based on the networks proposed in previous studies (Fang et al., 2020; Wu et al., 2024). The overall architecture of the proposed model is illustrated in Figure 5. Both tasks share a common backbone network for feature extraction, while task-specific prediction heads are employed to generate the corresponding outputs. In general, the backbone accounts for the majority of the model parameters in neural networks that process images or point clouds. Therefore, sharing the backbone enables both tasks to be performed while reducing the computational resources required for inference. Furthermore, although the proposed model takes point cloud data as input, only the three-dimensional coordinates of each point are used, while RGB information is excluded to improve robustness against variations in object texture and illumination.

**FIGURE 5:**
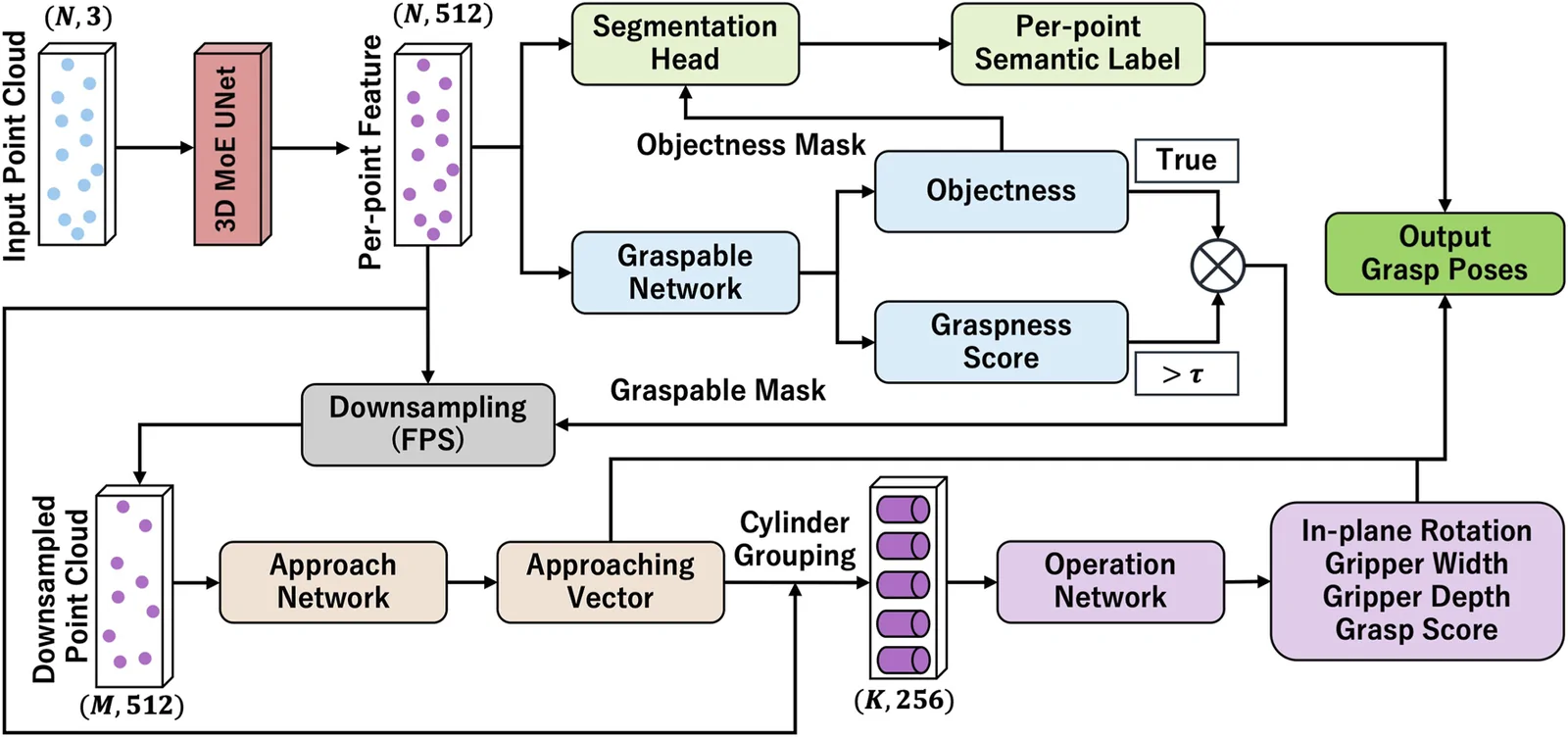
Overall architecture of the proposed multi-task model for 6-DOF grasp pose estimation and semantic segmentation.

As the backbone of the proposed model, we propose a 3D MoE UNet based on the MinkUNet14 architecture implemented with the Minkowski Engine (Choy et al., 2019; Ronneberger et al., 2015), incorporating a Mixture of Experts (MoE) structure (Shazeer et al., 2017). The use of MoE increases the representation capacity of the shared backbone while limiting the computational cost during inference. A straightforward approach to improving the representation capacity of a conventional 3D UNet is to increase its depth or width. However, this also increases the number of active parameters and the computational cost. In contrast, MoE employs multiple parallel experts and activates only a subset of them through sparse routing. This allows the model to increase its overall capacity without activating all parameters simultaneously, making it suitable for the proposed multi-task framework under limited computational resources.

The architecture of the proposed 3D MoE UNet and the output dimensions of each layer are illustrated in Figure 6. Specifically, the two deepest residual blocks in the encoder are replaced with residual MoE blocks which contain MoE layers. In shallow layers, low-level features such as local geometric structures and edges are extracted, resulting in relatively small variations in feature representations. In contrast, deeper layers capture high-level features through a larger receptive field, including the overall object geometry and surrounding scene context, leading to greater diversity in feature representations. Therefore, assigning feature extraction to multiple experts in these deeper layers is expected to improve the model’s ability to represent diverse object geometries and surrounding environments.

**FIGURE 6:**
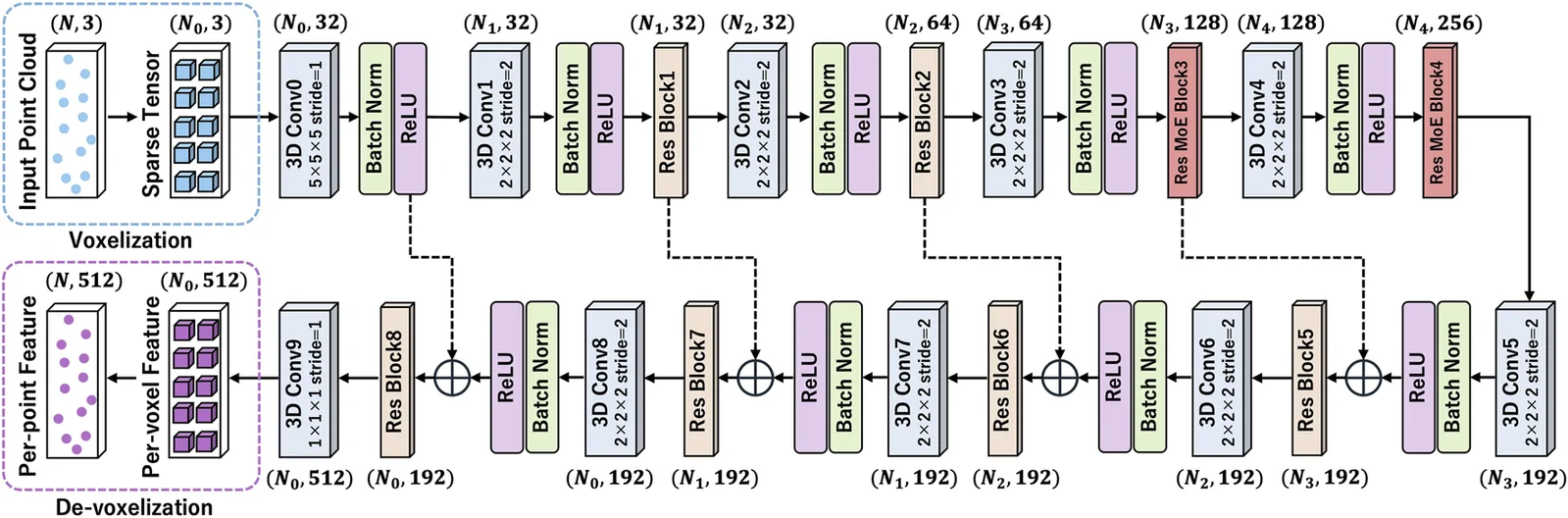
Overall architecture of the proposed 3D MoE UNet backbone.

The architecture of the residual MoE block is illustrated in Figure 7. Specifically, the first convolutional layer in a conventional residual block is expanded into multiple experts, each consisting of two convolutional layers. The gating layer selects the top- K experts, and the output of the MoE layer is computed as the weighted sum of their outputs. The output $y_{i}$ of the MoE layer for the i -th voxel is given by Equations 16, 17:

$$y_{i}=\underset{e\in T_{i}}{\sum }\alpha_{i,e}F_{e}\left(x_{i}\right)$$ (16)

$$\alpha_{i,e}=\frac{\exp\left(g_{i,e}\right)}{\sum_{j\in T_{i}}\exp\left(g_{i,j}\right)}\left(e\in T_{i}\right)$$ (17)

where $x_{i}$ denotes the feature vector of the i -th voxel, $F_{e}$ denotes the e -th expert, $\alpha_{i,e}$ is the weight assigned to the e -th expert, $T_{i}$ is the set of experts selected by the gating layer, and $g_{i,e}$ and $g_{i,j}$ are the logits produced by the gating layer. Furthermore, to prevent the expert collapse problem, in which the gating layer excessively concentrates on a subset of experts during training, the load balancing loss proposed in a previous study (Lepikhin et al., 2021) is introduced as an auxiliary loss. Let $p_{e}$ denote the average routing probability of the e -th expert and $f_{e}$ denote the fraction of inputs assigned to the e -th expert. The auxiliary loss is computed using Equation 18:

$$L_{\mathrm{aux}}=E\overset{E}{\underset{e=1}{\sum }}p_{e}f_{e}$$ (18)

where E denotes the number of experts. In our implementation, the number of experts is set to 8, and the gating layer is configured to select the top-2 experts.

**FIGURE 7:**
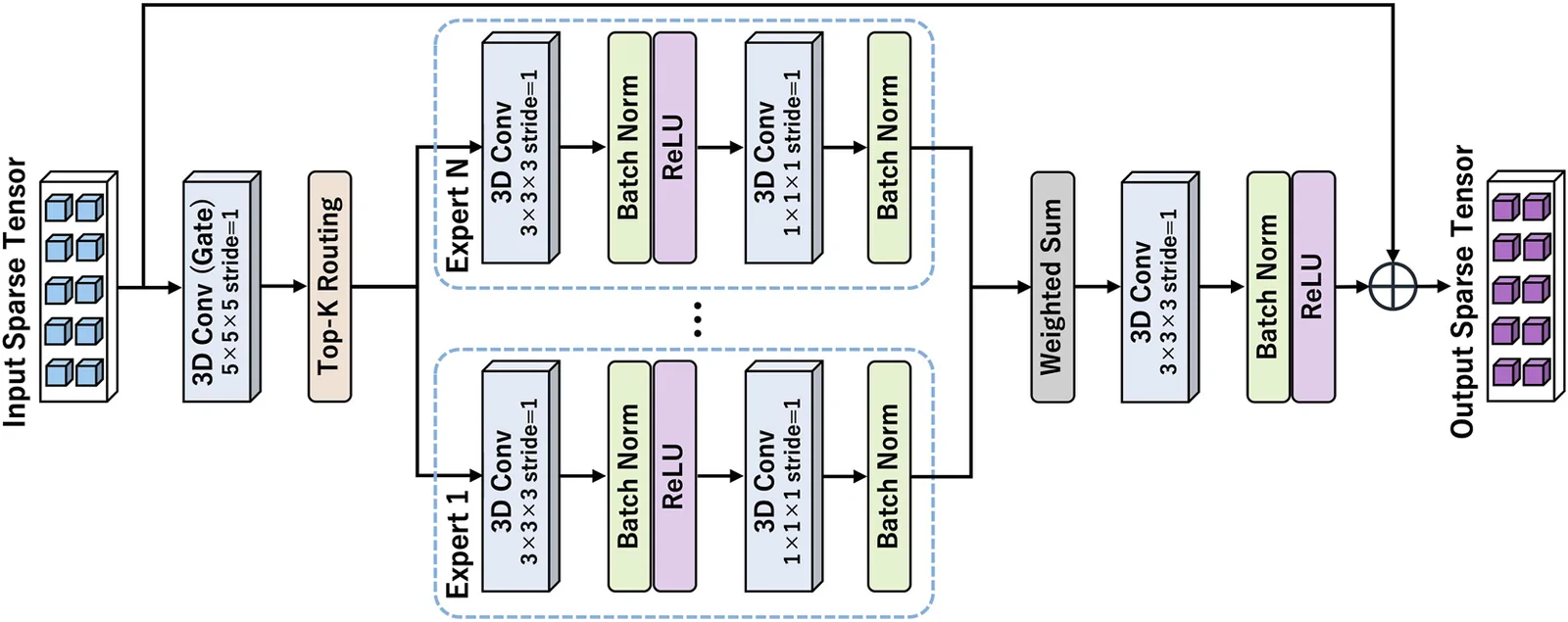
Overall architecture of the proposed residual MoE block.

In the Graspable Network, objectness prediction and graspness score (Wang et al., 2021) prediction are performed for each point. Objectness prediction performs binary classification to determine whether each point belongs to an object or the background. The graspness score represents the likelihood that a stable grasp can be achieved in the local neighborhood of a point. Points that belong to an object and have a graspness score greater than the predefined threshold are regarded as graspable points. These graspable points are then downsampled using the Farthest Point Sampling (FPS) algorithm (Eldar et al., 1994), and the resulting point set is used as the set of grasp candidate points. In our implementation, the threshold for the graspness score is set to 0.1, and the number of grasp candidate points after downsampling was set to 1024. As the loss functions, cross-entropy loss is employed for objectness prediction, yielding the objectness loss $L_{\mathrm{obj}}$ , while smooth L1 loss (Girshick, 2015) is employed for graspness score prediction, yielding the graspness loss $L_{\mathrm{grasp}}$ .

In the Segmentation Head, the objectness predicted by the Graspable Network is used to mask out background points from the input point cloud, and the remaining points are classified into their corresponding semantic categories. Cross-entropy loss is employed, yielding the segmentation loss $L_{\mathrm{seg}}$ . Label smoothing is applied to mitigate overfitting, with the smoothing coefficient set to 0.1.

In the Approach Network, the approaching vector, which represents the approach direction during grasping, is estimated for each grasp candidate point. Following the previous work (Fang et al., 2020), the Fibonacci sphere sampling algorithm (González, 2009) is employed to uniformly sample 300 points on the surface of a unit sphere centered at each grasp candidate point. The vectors from the sampled points to the sphere center are treated as candidate approaching vectors, and a score is predicted for each of the 300 candidate approaching vectors corresponding to each grasp candidate point. Smooth L1 loss is adopted, yielding the approaching vector loss $L_{\mathrm{app}}$ .

Subsequently, cylinder grouping is performed based on the approaching vector estimated by the Approach Network to extract local region features with geometric constraints. Specifically, a local coordinate system is constructed by setting the grasp candidate point as the origin, the estimated approaching vector as the x-axis, and the rotation about the x-axis to 0° . The coordinates of all points in the point cloud are then transformed into the local coordinate system. Neighboring points located within a cylindrical region with a radius of 0.05 m and a height ranging from −0.02 m to 0.04 m , whose axis is aligned with the x-axis of the local coordinate system, are grouped together and a feature representation is extracted for each cylinder group.

In the Operation Network, the features of the cylinder group corresponding to each grasp candidate point are used as input to estimate the in-plane rotation, gripper width, gripper depth, and grasp score which represents the grasp quality. The estimation of the in-plane rotation, gripper depth, and grasp score is formulated as classification tasks, and cross-entropy loss is employed, yielding the corresponding loss terms $L_{\mathrm{rot}}$ , $L_{\mathrm{depth}}$ , and $L_{\mathrm{score}}$ , respectively. The estimation of the gripper width is formulated as a regression task, and smooth L1 loss is employed, yielding the gripper width loss $L_{\mathrm{width}}$ . Specifically, the in-plane rotation is discretized into 12 classes with an interval of 15° , covering the range from 0° to 165° . The gripper depth is classified into four classes corresponding to the candidate set D={0.01,0.02,0.03,0.04} m . Similarly, the grasp score is classified into six classes corresponding to the candidate set S={0,0.2,0.4,0.6,0.8,1} . The final grasp score is computed as the weighted sum of the score values of all classes, where the weights are given by the predicted probabilities output by model.

Based on the loss functions described above, the total loss function is defined in Equation 19:

$$\begin{array}{cc} L_{\mathrm{total}}= & \lambda_{\mathrm{aux}}L_{\mathrm{aux}}+\lambda_{\mathrm{obj}}L_{\mathrm{obj}}+\lambda_{\mathrm{grasp}}L_{\mathrm{grasp}}+\lambda_{\mathrm{seg}}L_{\mathrm{seg}}+\lambda_{\mathrm{app}}L_{\mathrm{app}}\\ & +\lambda_{\mathrm{rot}}L_{\mathrm{rot}}+\lambda_{\mathrm{depth}}L_{\mathrm{depth}}+\lambda_{\mathrm{score}}L_{\mathrm{score}}+\lambda_{\mathrm{width}}L_{\mathrm{width}} \end{array}$$ (19)

where λ denotes the weighting coefficient for each loss term. The weighting coefficients are set to $\lambda_{\mathrm{aux}}=0.05$ , $\lambda_{\mathrm{seg}}=0.5$ , $\lambda_{\mathrm{grasp}}=10$ , $\lambda_{\mathrm{app}}=100$ , and $\lambda_{\mathrm{width}}=10$ , while all other weighting coefficients are set to 1. These coefficients were empirically selected to balance the relative contributions of the individual loss terms by bringing their magnitudes to approximately the same order during training.

Finally, a 6-DOF grasp pose is constructed from the three-dimensional coordinates of each grasp candidate point and the estimated approaching vector and in-plane rotation. The gripper width, gripper depth, grasp score, and the object class predicted by the Segmentation Head for each grasp candidate point are then integrated with the grasp pose to generate the final grasp representation.

## 4 Experiments

### 4.1 Model performance evaluation

The proposed model was implemented using the PyTorch library (Paszke et al., 2019) and was trained and evaluated on the GraspNet-1Billion dataset (Fang et al., 2020).^1^ The source code, configuration files, and scripts required to reproduce the experiments are publicly available at the repository specified in the Data Availability Statement.

GraspNet-1Billion is a large-scale real-world benchmark dataset for general object grasping and is designed not only for grasp pose estimation but also for various vision tasks, including 6-DOF object pose estimation and unseen object segmentation. The dataset contains 97,280 RGB-D images of 190 cluttered scenes comprising 88 general objects, which were captured separately using the Azure Kinect and the RealSense D435 RGB-D cameras. Each image is annotated with dense 6-DOF grasp pose labels, resulting in more than 1.1 billion grasp pose annotations across the entire dataset. Of the 190 scenes, 100 constitute the training set and the remaining 90 constitute the test set. The test set is further divided equally into seen, similar, and novel subsets, containing objects present in the training set, objects similar to those in the training set, and objects never seen during training, respectively. Furthermore, Wu et al. (2024) extended the GraspNet-1Billion dataset by introducing graspness score (Wang et al., 2021) labels and proposed a data preprocessing method that selects only representative and high-quality grasp pose candidates from the original grasp annotations. This approach was reported to improve model performance while reducing training time, memory consumption, and storage requirements to approximately 1/4, 1/8, and 1/30 of the original, respectively.

Based on the above, we preprocessed the GraspNet-1Billion dataset using the method proposed by Wu et al. (2024) for the model evaluation experiments conducted in this study. In addition, many of the 88 objects in the GraspNet-1Billion dataset exhibit similar geometric shapes, making it difficult to distinguish between objects when only three-dimensional coordinates of point cloud are used. Therefore, we reclassified the objects into seven categories based on their geometric characteristics: pump bottle, tube, container, irregular-shaped object, spherical object, elongated/cylindrical object, and cuboid object. A new category label was then assigned to each point in the point cloud and used for training the semantic segmentation task. The mapping of the 88 GraspNet objects to the seven semantic categories is provided in Supplementary Table S1. The class distribution of the training set, including the number of objects and the number and proportion of labeled points in each category, is summarized in Supplementary Table S2.

Following the previous study (Fang et al., 2020), average precision (AP) was adopted as the evaluation metric for the grasp pose estimation task. For each of the top- K predicted grasp poses, grasp stability was determined based on the force closure metric (Nguyen, 1988). With a given friction coefficient μ , a predicted grasp pose was regarded as a true positive if stable grasping was achievable with a minimum required friction coefficient no greater than μ . For each friction coefficient μ , ${AP}_{\mu }$ was computed as the average precision over K=1 to 50. The final AP was then calculated by averaging ${AP}_{\mu }$ over μ∈{0.2,0.4,0.6,0.8,1.0,1.2} .

For the semantic segmentation task, mean intersection over union (mIoU) was used as the evaluation metric. Let x denote the number of classes and $N_{ij}$ denote the number of points whose ground-truth class is i and that are classified as class j , the mIoU is computed using Equation 20:

$$mIoU=\frac{1}{x}\overset{x}{\underset{i=1}{\sum }}\frac{N_{ii}}{\sum_{i=1}^{x}N_{ij}+\sum_{j=1}^{x}N_{ji}-N_{ii}}$$ (20)

All experiments were conducted using a single NVIDIA GeForce RTX 4080 SUPER GPU. Training and evaluation were conducted separately using the data captured by Kinect and RealSense cameras in the GraspNet-1Billion dataset. The Adam optimizer (Kingma and Ba, 2014) was employed with an initial learning rate of $1e^{-3}$ and a minimum learning rate of $1e^{-5}$ . A cosine annealing learning rate scheduler was used to decay the learning rate at each epoch. The batch size was set to 4, and the model was trained for 40 epochs.

For comparison, the backbone of the proposed model was replaced with PointNet++ (Qi et al., 2017) and 3D UNet (Choy et al., 2019; Ronneberger et al., 2015) following previous studies (Fang et al., 2020; Wu et al., 2024), and the resulting models were evaluated. In addition to this backbone comparison within the proposed framework, a single-task model for grasp pose estimation was constructed by removing the segmentation head, and its performance was compared with representative previous methods (Fang et al., 2020; Wang et al., 2021; Liu et al., 2022; Wang et al., 2023; Wu et al., 2024). Among these studies, Fang et al. (2020) and Wang et al. (2021) both adopted PointNet++-based models, with the latter further introducing graspness prediction. Liu et al. (2022) employed a Point Transformer-based (Zhao et al., 2021) architecture, whereas Wang et al. (2023) proposed a graph-based model that constructs multi-level graphs from the input point cloud. Wu et al. (2024) adopted a 3D UNet-based model and introduced a preprocessing method for the GraspNet-1Billion dataset. The results for Wu et al. (2024) were obtained by evaluating their publicly available implementation in our experimental environment using the same parameter settings, whereas the results of the other methods were taken from their respective publications.

The evaluation results of the multi-task models for the grasp pose estimation task and the semantic segmentation task are shown in Tables 1,2, respectively. The comparison between the proposed single-task model and the methods from previous studies is shown in Table 3. For the multi-task models, the proposed 3D MoE UNet backbone improved the grasp pose estimation performance on the RealSense data by 2.43, 1.98, and 2.14 AP points on the seen, similar, and novel test sets, respectively, compared with the 3D UNet backbone. On the Kinect data, the proposed backbone achieved improvements of 5.24 and 2.25 AP points on the seen and similar test sets, respectively, whereas the AP on the novel test set decreased by 0.65 points. For the semantic segmentation task, the model using the proposed 3D MoE UNet achieved the best performance on all test sets except for the novel test set of the RealSense data. Furthermore, the proposed single-task model achieved the best grasp pose estimation performance among the compared methods across all test sets.

**TABLE 1:** Grasp pose estimation performance of multi-task models with different backbones on the RealSense/Kinect data.

Backbone	Seen			Similar			Novel		
	AP	${AP}_{0.8}$	${AP}_{0.4}$	AP	${AP}_{0.8}$	${AP}_{0.4}$	AP	${AP}_{0.8}$	${AP}_{0.4}$
PointNet++	61.78/56.54	74.05/67.74	53.70/48.16	53.72/44.13	66.19/54.86	43.26/33.28	21.92/15.95	27.31/19.86	10.02/7.65
3D UNet	63.91/57.11	74.91/68.58	58.93/49.40	55.23/45.59	67.21/55.93	46.99/36.60	21.90/**18.65**	27.12/**23.12**	11.27/9.71
3D MoE UNet	**66.34/62.35**	**76.03/73.19**	**61.29/56.51**	**57.21/47.84**	**69.05/58.51**	**49.91/38.56**	**24.04**/18.00	**29.00**/22.52	**12.15/9.78**

The best results are highlighted in bold, and the results of the proposed model are shaded in gray.

**TABLE 2:** Semantic segmentation performance on the RealSense/Kinect data.

Backbone	Seen	Similar	Novel
PointNet++	46.01/47.28	26.26/26.63	8.22/8.38
3D UNet	73.24/74.15	38.84/35.49	**13.30**/7.83
3D MoE UNet	**73.59/77.57**	**40.02/37.05**	11.92/**10.57**

The best results are highlighted in bold, and the results of the proposed model are shaded in gray.

**TABLE 3:** Comparison of grasp pose estimation performance between the proposed single-task model and existing methods on the RealSense/Kinect data.

Method	Seen			Similar			Novel		
	AP	${AP}_{0.8}$	${AP}_{0.4}$	AP	${AP}_{0.8}$	${AP}_{0.4}$	AP	${AP}_{0.8}$	${AP}_{0.4}$
Fang et al. (2020)	27.56/29.88	33.43/36.19	16.95/19.31	26.11/27.84	34.18/33.19	14.23/16.62	10.55/11.51	11.25/12.92	3.98/3.56
Liu et al. (2022)	39.81/35.97	47.54/41.69	36.42/31.86	29.32/29.71	34.80/35.67	25.19/24.19	13.83/11.41	17.11/14.42	7.67/5.84
Wang et al. (2023)	43.33/41.48	52.56/49.84	34.03/33.86	39.98/35.29	48.66/43.15	32.00/26.89	14.90/11.57	18.66/14.31	7.76/5.24
Wang et al. (2021)	65.70/61.19	76.25/71.46	61.08/56.04	53.75/47.39	65.04/56.78	45.97/40.43	23.98/19.01	29.93/23.73	**14.05**/10.60
Wu et al. (2024)	67.62/60.47	78.46/70.98	63.37/54.95	59.98/48.77	71.94/59.23	53.25/40.66	23.68/17.58	29.27/21.95	12.74/9.94
Ours	**68.42/62.63**	**79.38/73.61**	**64.22/56.87**	**60.57/50.14**	**72.94/60.77**	**53.39/41.73**	**24.38/19.15**	**30.05/23.88**	12.73/**10.61**

The best results are highlighted in bold, and the results of the proposed model are shaded in gray.

To further analyze the semantic segmentation performance of the proposed model, we computed the per-class IoU and row-normalized confusion matrices, which are presented in Supplementary Table S3 and Supplementary Figures S2 and S3, respectively. Although relatively high IoU values were obtained for most categories in the seen test set, the performance decreased markedly for several categories in the similar and novel test sets. In particular, pump bottle, container, spherical object, elongated/cylindrical object, and cuboid object showed low IoU values for one or both camera test sets. The confusion matrices indicate that these decreases were associated with systematic inter-class confusion, such as cuboid objects being classified as container and pump bottle objects being confused with tube. This is likely because some similar and novel objects differ in shape from the training samples of the same category while sharing similar geometric characteristics with objects from other categories, making them more difficult to distinguish. In addition, the differences between the results for the RealSense and Kinect data may be attributed to sensor-specific characteristics of the point clouds.

Figure 8 shows the selection frequency of each expert in the two residual MoE blocks during the evaluation of the multi-task model using the proposed 3D MoE UNet. Across all test sets, the selection frequencies of the experts were well balanced, with no significant bias toward any particular expert. These results indicate that expert collapse did not occur and that all experts contributed to the inference process in a balanced manner.

**FIGURE 8:**
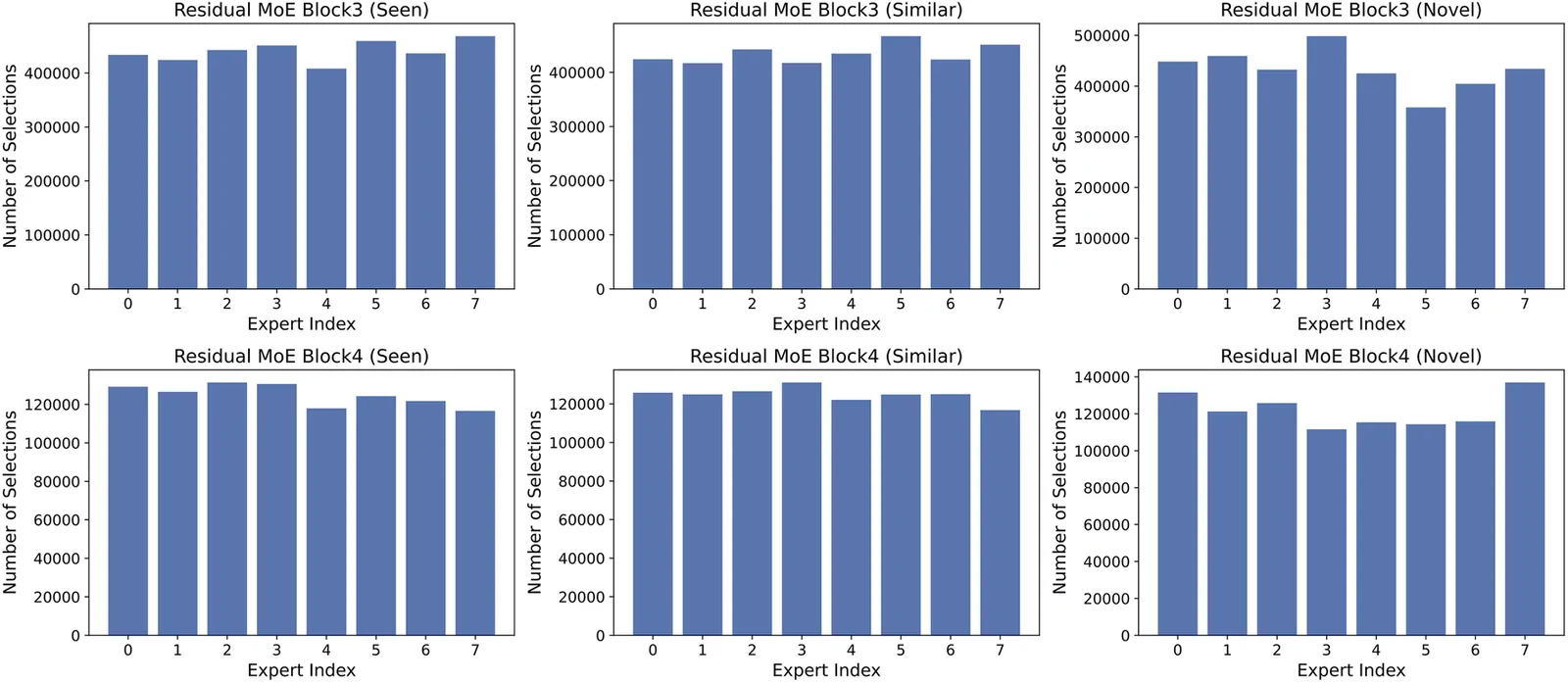
Expert selection counts of the two residual MoE blocks on the seen, similar, and novel test sets.

To investigate how different MoE configurations affect model performance, ablation experiments were conducted on the RealSense data by varying the number of experts, Top-K routing, the number and placement of the MoE blocks, and the use of the load-balancing loss. The results for grasp pose estimation and semantic segmentation are summarized in Supplementary Tables S4 and S5, respectively. Although the performance differences among the tested configurations were relatively small, several tendencies could still be observed. Top-1 routing, fewer experts, and fewer MoE blocks tended to improve performance on the seen test set, whereas Top-4 routing, more experts, or different block placements showed advantages on the similar or novel test sets. In addition, as shown in Supplementary Figure S4, removing the load-balancing loss resulted in strongly imbalanced expert utilization. While some alternative configurations achieved higher values on individual test sets, no configuration consistently outperformed the adopted setting across both tasks. These results suggest that the adopted configuration provides a reasonable balance in performance across the different test sets while maintaining balanced expert utilization, although the MoE configuration may still have room for further optimization.

The decrease of 0.65 AP points on the novel test set of Kinect data was relatively small and the degradation was not observed for all evaluation metrics, as ${AP}_{0.4}$ slightly increased. Since all the evaluated models generally performed worse on the Kinect data than on the RealSense data, sensor-specific characteristics of the point clouds in Kinect data including depth noise, missing points, and variations in point density, may have made it more difficult to extract stable geometric features. This may have particularly limited generalization to previously unseen object geometries. For semantic segmentation, the mIoU on the novel test set of RealSense data decreased by 1.38 points compared with that of the model using 3D UNet backbone. Since the AP of grasp pose estimation increased by 2.14 points on the same test set, this contrasting result may indicate a trade-off in the shared feature representation between the two tasks. Although the proposed backbone exhibited a slight performance degradation on a small number of test sets, the decrease was limited. Moreover, it achieved improved performance on all remaining test sets, demonstrating its overall effectiveness in enhancing both grasp pose estimation and semantic segmentation.

Compared with the single-task model, the multi-task model generally showed lower grasp pose estimation performance. However, the multi-task model using the proposed 3D MoE UNet achieved grasp pose estimation results that were closer to those of the single-task model based on the 3D UNet proposed by Wu et al. (2024). These results indicate that the proposed method effectively incorporates semantic segmentation within a single model while preserving grasp pose estimation performance to the greatest extent possible.

Furthermore, the proposed multi-task model contains 24.2 M parameters in total, and since only the top-2 experts are activated during inference, the number of active parameters is 17.1 M. In comparison, the single-task model based on the 3D UNet contains 15.8 M parameters, indicating that the proposed model increases the number of active parameters by only approximately 1.3 M. By contrast, since the 3D UNet backbone used in the single-task model contains 14.7 M parameters, introducing a separate semantic segmentation model with the same backbone would require at least an additional 14.7 M active parameters during inference. These results demonstrate that the proposed multi-task model substantially reduces the computational resources required compared with employing an independent semantic segmentation model.

### 4.2 Real-world grasping experiments

In the real-world grasping experiments, the proposed control system was implemented on the coupled tendon-driven 7-DOF robotic arm described in Section 3.1. As shown in Figures 9a, 10 general objects were selected to include variations in shape, size, and surface properties that may affect grasp pose estimation and grasp execution. Among the 10 target objects, objects 1, 2, and 3 were seen objects included in the training data, whereas the remaining seven were unseen objects, allowing the experiments to evaluate both grasping performance on familiar objects and generalization to previously unseen objects. For each object, the robotic arm performed 10 pick-and-place trials in a cluttered environment, and the number of successful trials was recorded. The clutter level was maintained at a minimum of 4 objects per scene, including the target object, while the exact number of objects and their positions and orientations were manually varied between trials to create different scene configurations within the reachable area of the robotic arm. No fixed quantitative constraints were imposed on the object positions, orientations, or inter-object distances. A trial was considered successful when the robotic arm grasped the specified object without causing it to topple, lifted it from the table, transported it to the predefined placement position, and released it without dropping the object.

**FIGURE 9:**
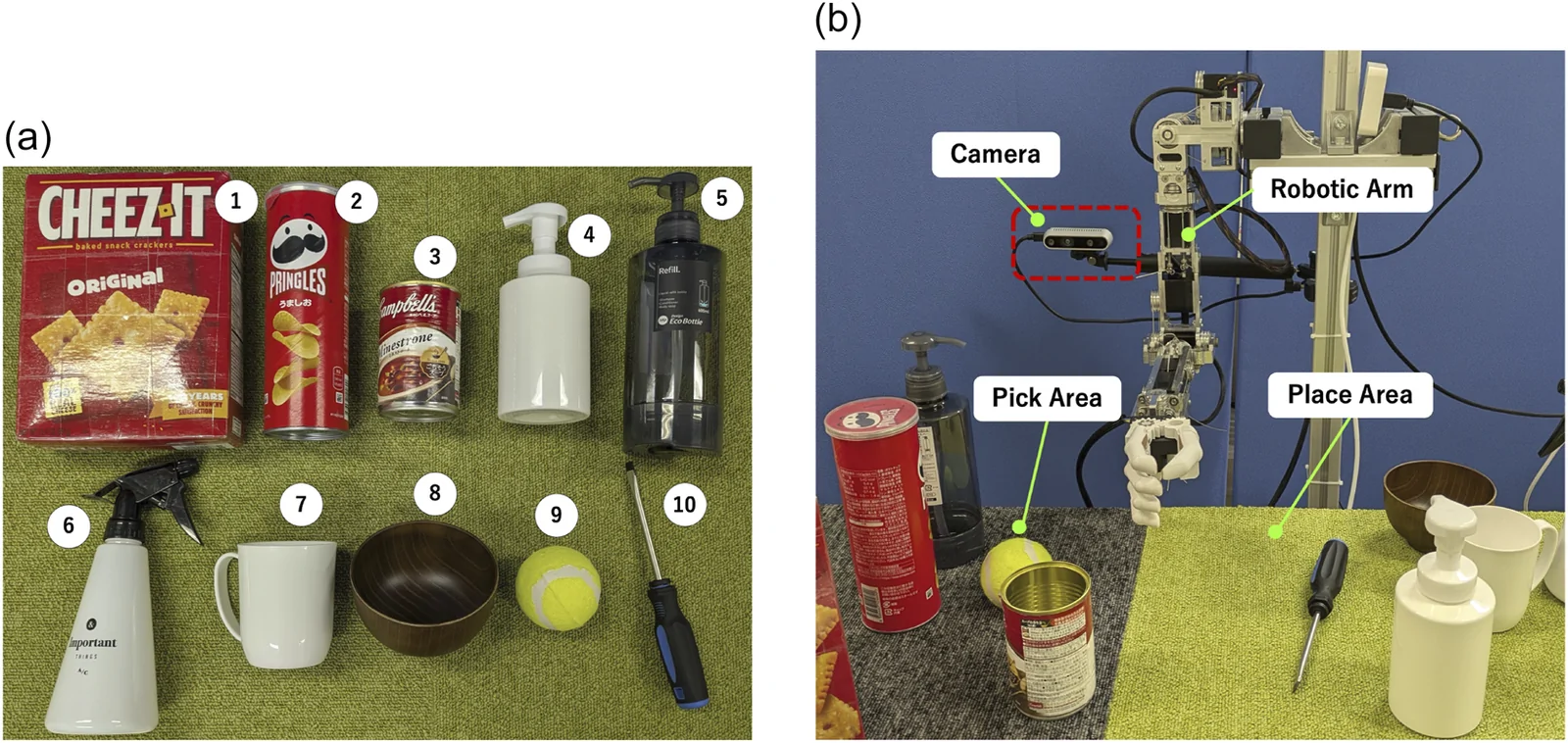
Objects and experimental setup used in the real-world grasping experiments. **(a)** Objects used in the experiments. **(b)** Experimental setup.

**FIGURE 10:**
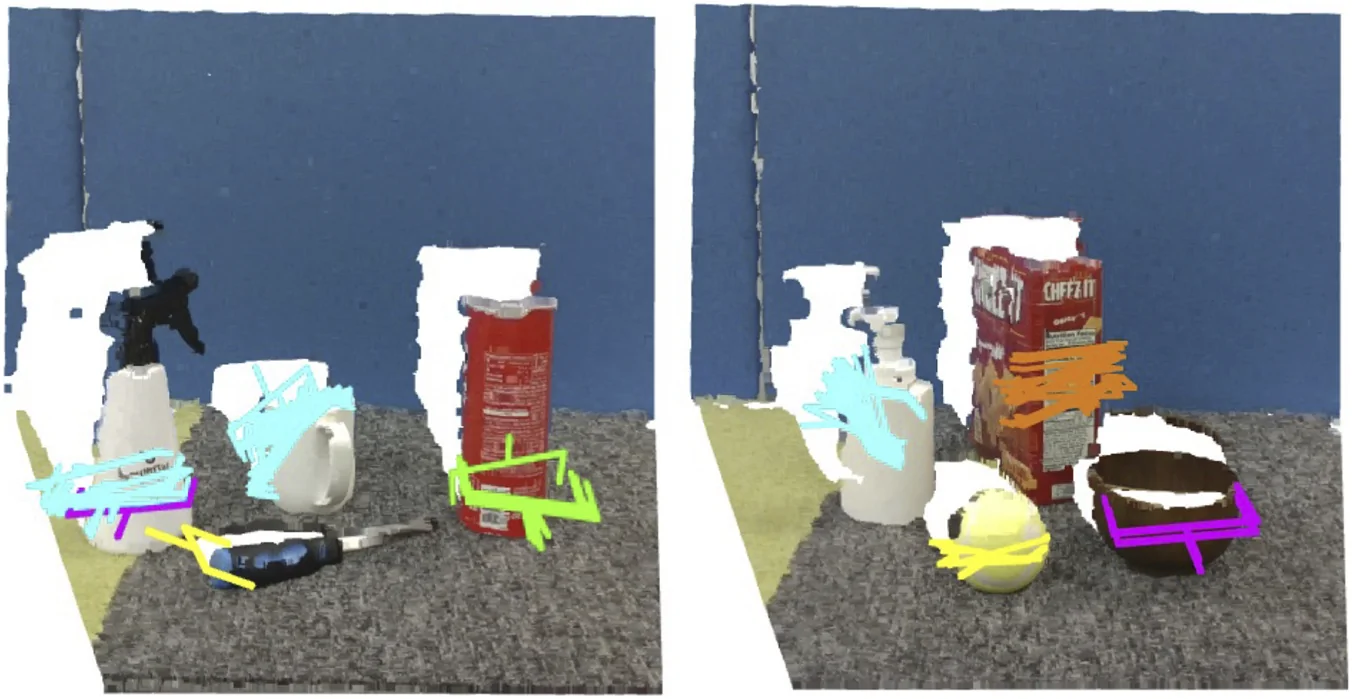
Example visualization of 6-DOF grasp pose estimation results in a cluttered scene.

The control system was implemented using ROS 2 Humble (Macenski et al., 2022) on a PC running Ubuntu 22.04, and the inverse kinematics solver was implemented as described in Section 3.2. To generate the trajectory toward the target end-effector pose, linear interpolation was applied to the position and spherical linear interpolation (Shoemake, 1985) to the orientation. The calculated target joint angles were transmitted to the motors of the robotic arm via RS-485 communication.

The control procedure is as follows. First, the depth image of a cluttered scene captured by an RGB-D camera is converted into point-cloud data, and grasp poses are estimated using the model proposed in Section 3.3. The estimated grasp poses are transmitted to the robotic arm control system via TCP communication. The target object is then manually selected by choosing the corresponding category in a graphical user interface, and the grasp pose with the highest grasp score among those associated with the selected object is used to execute the grasping motion. To avoid collisions with the target object and enable a stable approaching motion, a pre-grasp pose is defined by offsetting the selected grasp pose by 0.1 m in the direction opposite to its approaching vector. The end-effector first moves to the pre-grasp pose, then advances to the grasp pose to grasp the target object and lifts the object vertically upward. Finally, the end-effector moves to a predefined target pose for the placing motion and releases the object at the designated location.

The experimental setup is shown in Figure 9b. An Intel RealSense Depth Camera D435i was used as the RGB-D camera. An example visualization of the estimated 6-DOF grasp poses in a cluttered scene is presented in Figure 10. The different colors of the grasp poses represent different semantic categories in the semantic segmentation results. The execution of the pick-and-place operation is illustrated in Figure 11, and the experimental results are summarized in Table 4.

**FIGURE 11:**
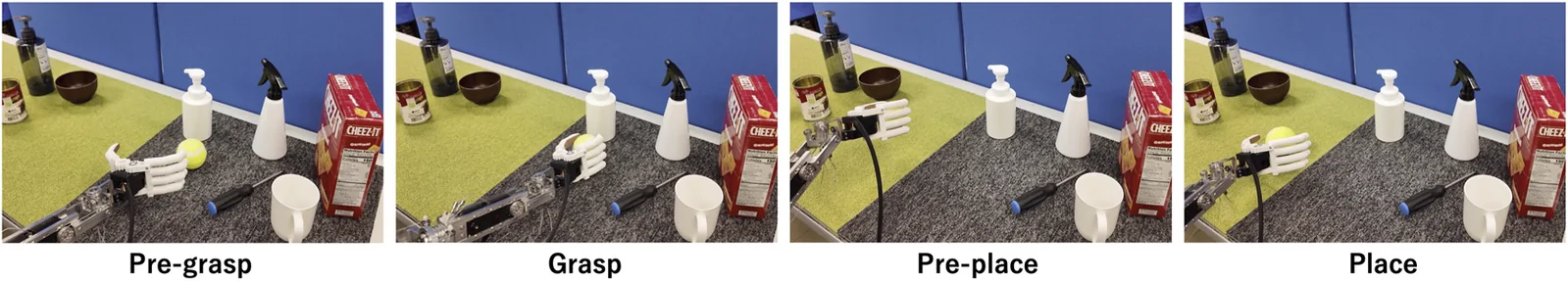
Execution sequence of the pick-and-place operation.

**TABLE 4:** Results of the real-world pick-and-place experiments for the 10 target objects.

Object	Seen/Unseen	Successes/Trials	Success rate (%)
1	Seen	10/10	100.0
2	Seen	10/10	100.0
3	Seen	10/10	100.0
4	Unseen	9/10	90.0
5	Unseen	4/10	40.0
6	Unseen	9/10	90.0
7	Unseen	7/10	70.0
8	Unseen	1/10	10.0
9	Unseen	7/10	70.0
10	Unseen	0/10	0.0
Seen objects	–	30/30	100.0
Unseen objects	–	37/70	52.9
Overall	–	67/100	67.0

The experimental results showed that the pick-and-place success rate was 100% for all three seen objects. Among the unseen objects, objects 4, 6, 7, and 9 achieved success rates of at least 70%, whereas objects 5, 8, and 10 exhibited relatively low success rates. One possible reason for the low success rate of object 5 is its translucent plastic material. Because the RealSense D435i estimates depth using infrared stereo images, the transmission and refraction of infrared light by translucent materials can interfere with stereo matching, resulting in missing or inaccurate depth measurements. Consequently, portions of the target object’s point cloud may be missing, which can prevent reliable grasp pose estimation. In addition, the robotic gripper used in this study has only one degree of freedom and therefore has limited adaptability to object geometry compared with multi-DOF robotic hands, restricting the contact locations at which stable grasps can be achieved. For objects with limited stable grasping regions, such as objects 8 and 10, or for objects with small dimensions, it can therefore be difficult to obtain sufficient contact for a stable grasp. Moreover, this limitation also makes it difficult to avoid collision with the supporting surface during grasping. The above factors likely contributed to the observed grasp failures.

Furthermore, in the real-world grasping experiments, the proposed model operated in real time at an average processing speed of 20 FPS, and the overall memory usage of the system including the robotic arm control system was 5.1 GB of RAM and 3.12 GB of VRAM. The measured memory requirements suggest that the proposed system could be deployed on a compact edge computer, such as the Jetson Orin^2^, and integrated into a standalone assistive robot. However, its real-time performance on such hardware remains to be experimentally verified.

## 5 Conclusion

In this study, with a view to applications in assistive robots, we proposed an end-to-end multi-task model that simultaneously performs 6-DOF grasp pose estimation and semantic segmentation for multiple general objects in cluttered environments. We also introduced a geometry-based inverse kinematics method for a coupled tendon-driven 7-DOF robotic arm, enabling stable real-time motion control.

The performance of the proposed model was evaluated using the GraspNet-1Billion dataset, a benchmark dataset for general object grasping. The experimental results demonstrated that the proposed 3D MoE UNet improved the performance of both grasp pose estimation and semantic segmentation compared with models employing PointNet++ or a conventional 3D UNet. In addition, the proposed multi-task model incorporated semantic segmentation while maintaining grasp pose estimation performance comparable to that of a conventional single-task model.

In the real-world grasping experiments, the proposed model was integrated into the control system of the 7-DOF robotic arm, and the success rates of pick-and-place operations were evaluated using 10 general objects in cluttered environments. The results showed that the proposed system achieved high success rates not only for seen objects but also for several novel objects, demonstrating its effectiveness in real-world environments. Furthermore, the proposed model operated in real time with limited computational resources, indicating its potential for deployment on standalone assistive robots.

Nevertheless, several limitations were also identified. The adopted MoE architecture increases the total number of model parameters and introduces additional complexity in expert routing, while its configuration involves additional design choices, such as the number of experts and the routing strategy. Accurate depth information could not be obtained for translucent objects, and the grasping stability of the robotic gripper decreased when handling objects with complex shapes or small objects for which only a limited number of stable grasping positions were available. In addition, since target object selection in the current system is based on semantic segmentation into seven categories, multiple objects belonging to the same category cannot be uniquely distinguished based solely on the segmentation results.

Future work may therefore include further optimization of the MoE configuration to improve the balance between model performance and computational efficiency. A robust point cloud acquisition method for translucent objects and a multi-DOF robotic hand with greater adaptability to objects with complex shapes and small dimensions should also be investigated. In addition, instance segmentation should be incorporated to distinguish individual object instances within the same semantic category. Furthermore, toward the practical use of the proposed system on an assistive robot, the method for specifying the target object should be extended to enable easy operation by users with limited physical mobility. This may involve introducing intuitive interfaces based on voice input or electromyographic (EMG) signals.

## Data Availability

Publicly available datasets were analyzed in this study. This data can be found here: https://graspnet.net/. The code required to reproduce the experiments is available at https://github.com/TogoLab/seg_grasp.

## Footnotes

1 GraspNet-1Billion dataset: https://graspnet.net/

2 NVIDIA Jetson Orin Series: https://www.nvidia.com/en-us/autonomous-machines/embedded-systems/jetson-orin/
