# Supplementary material for: Development of a 7-DOF robotic arm control system for autonomous grasping of user-specified general objects
Source: Front Robot AI. 2026 Oct 21;13:1955232. doi: 10.3389/frobt.2026.1955232 (PMC13730867; doi:10.3389/frobt.2026.1955232)
Supplement: Supplementary file 1 [file DataSheet1.pdf]

Supplementary Material

1 FIGURES

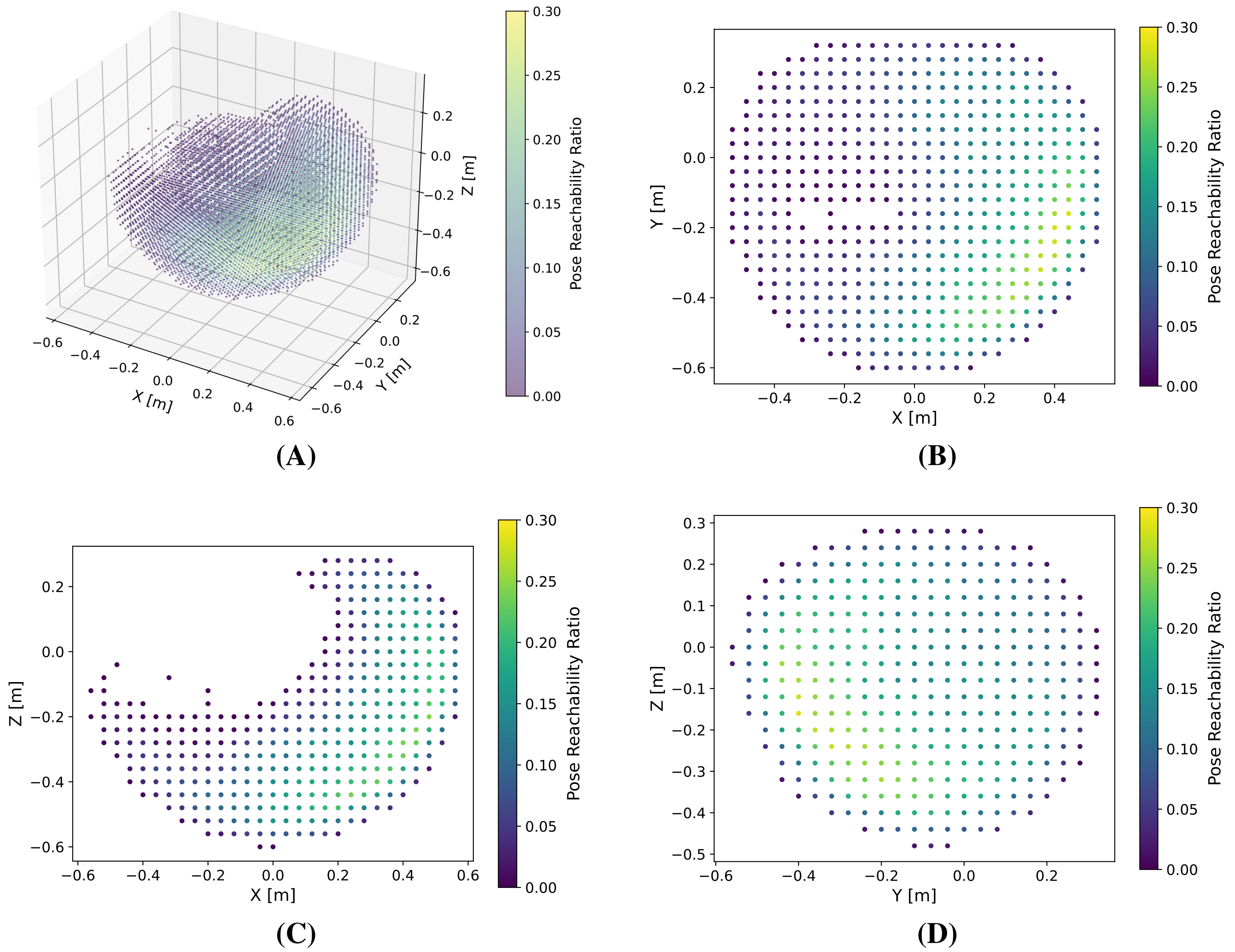

Figure S1. Reachable pose workspace of the robotic arm. **(A)** Three-dimensional distribution of the pose reachability ratio. Cross-sectional distributions in the **(B)** XY plane at $z = -0.25\,\mathrm{m}$, **(C)** XZ plane at $y = -0.078\,\mathrm{m}$, and **(D)** YZ plane at $x = 0.356\,\mathrm{m}$.

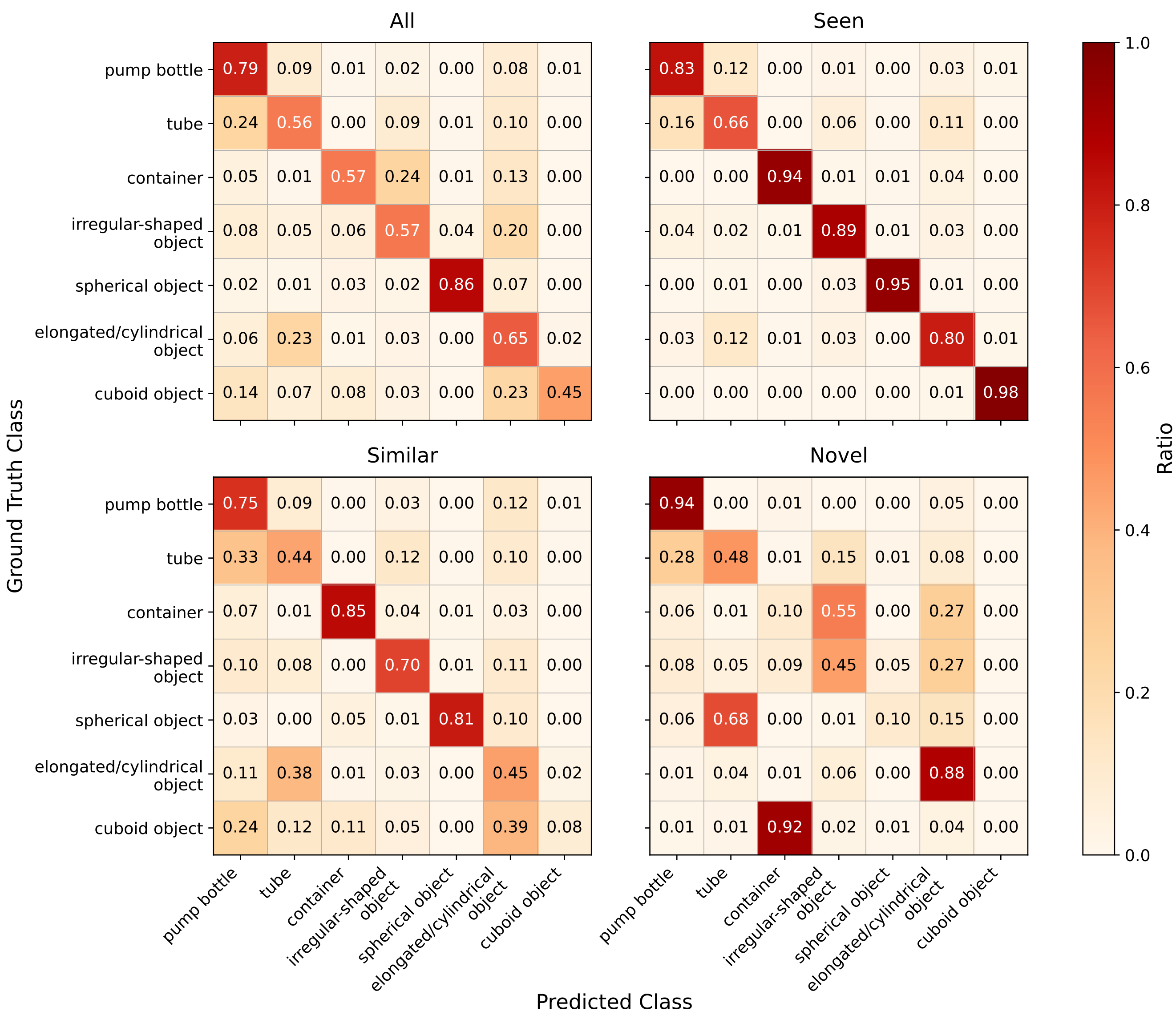

Figure S2. Confusion matrices of the semantic segmentation results on the RealSense test sets.

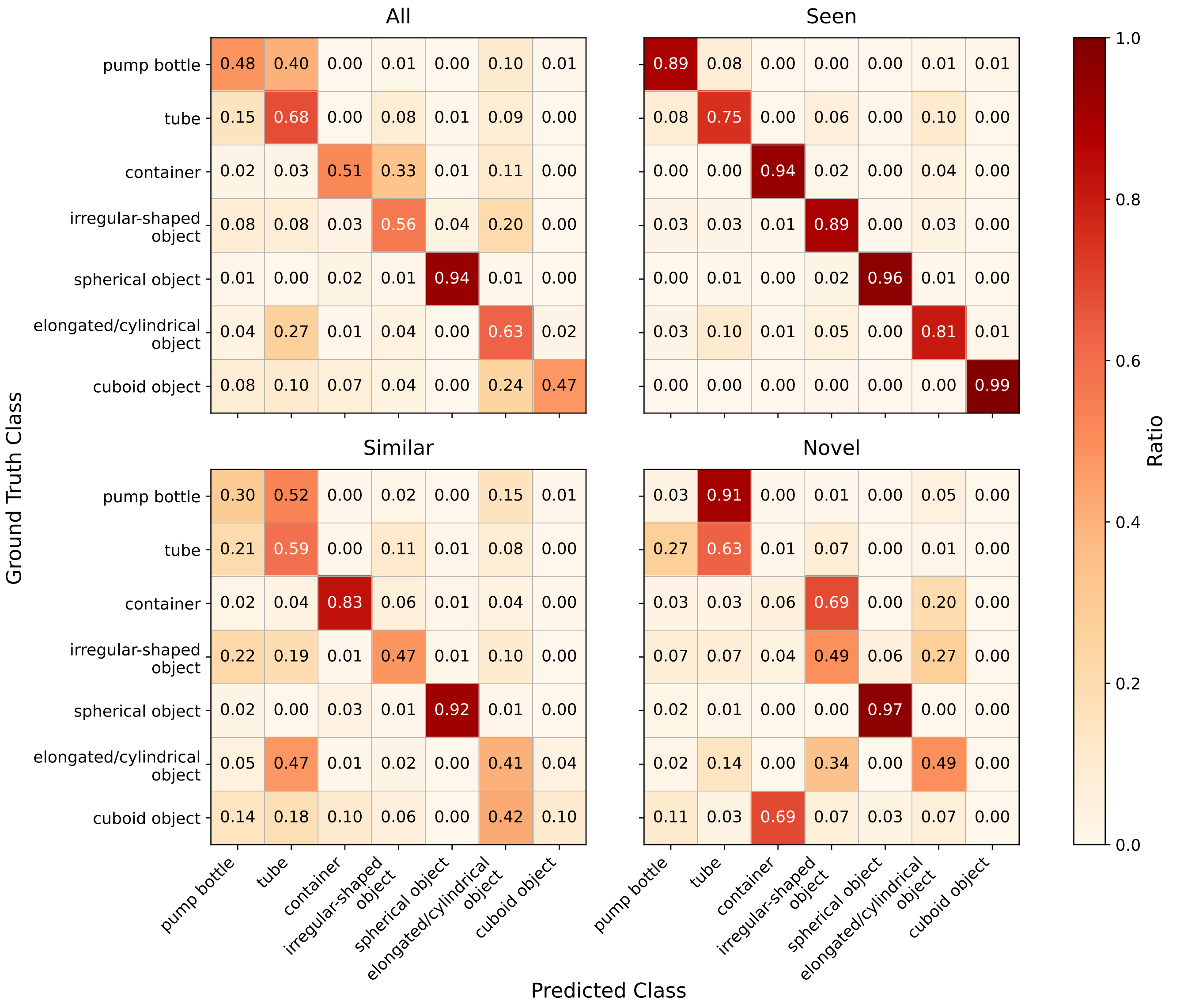

Figure S3. Confusion matrices of the semantic segmentation results on the Kinect test sets.

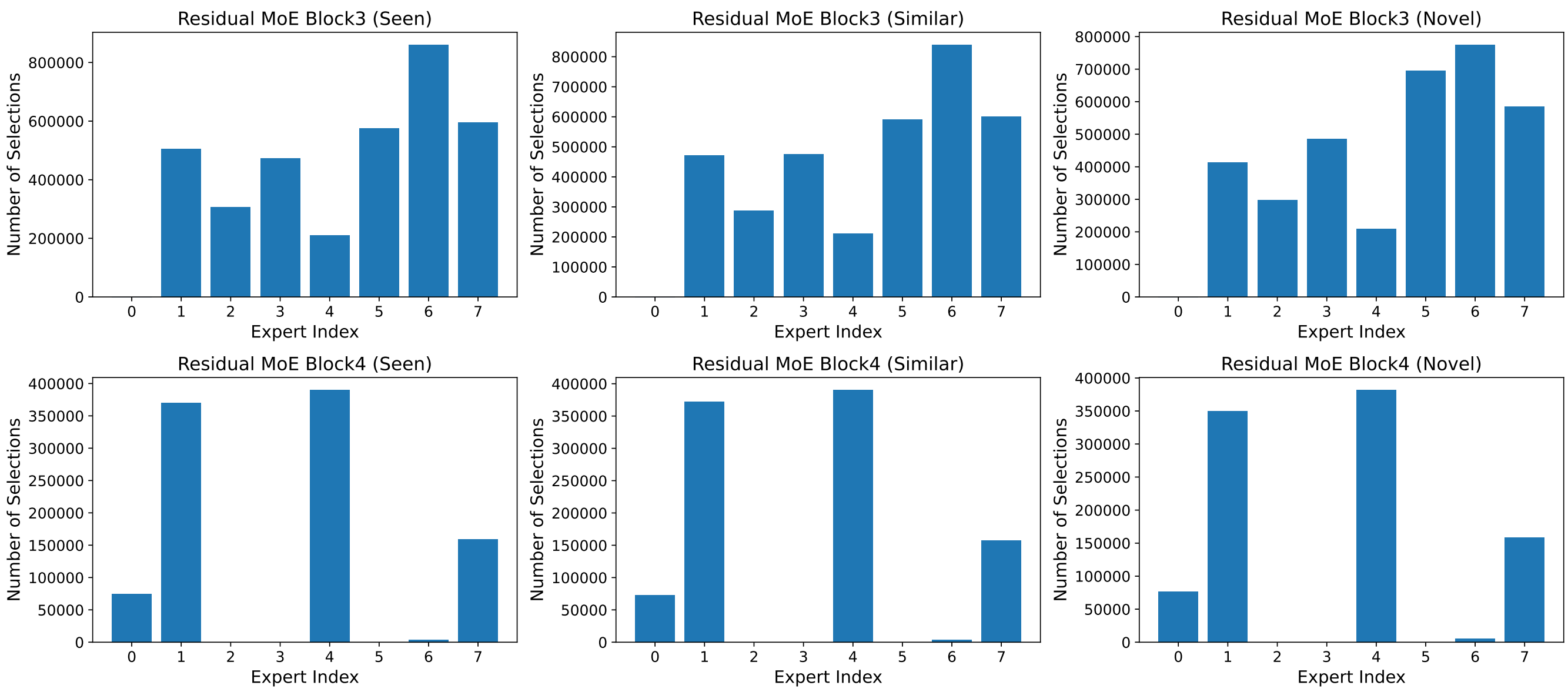

Figure S4. Expert selection counts of the two residual MoE blocks on the seen, similar, and novel test sets in the ablation experiment without the load-balancing loss.

2 TABLES

Table S1. Mapping of the 88 GraspNet objects into seven semantic categories.

Category	GraspNet object IDs
pump bottle	3, 36, 37, 38, 40, 44
tube	35, 41, 42, 47, 59, 60, 62, 63, 65, 66
container	6, 7, 22, 23, 46, 74
irregular-shaped object	8, 24, 25, 26, 27, 28, 29, 30, 31, 32, 33, 45, 48, 49, 50, 51, 52, 53, 54, 55, 56, 67, 69, 70, 71, 72, 73, 75, 76, 77, 78, 79, 80, 81, 82, 83, 84, 85, 86, 87
spherical object	11, 12, 13, 14, 15, 16, 17, 21
elongated/cylindrical object	2, 5, 9, 10, 18, 19, 20, 34, 43, 57, 58, 61, 64
cuboid object	0, 1, 4, 39, 68

Table S2. Class distribution of the seven semantic categories in the RealSense and Kinect training sets.

Category	No. of objects	No. of labeled points		Proportion (%)	
		RealSense	Kinect	RealSense	Kinect
pump bottle	6	31,379,254	25,265,348	13.92	14.75
tube	10	34,958,606	26,158,852	15.51	15.27
container	6	31,576,208	24,669,520	14.01	14.40
irregular-shaped object	40	40,079,434	29,092,977	17.78	16.99
spherical object	8	10,583,113	8,165,253	4.69	4.77
elongated/cylindrical object	13	49,474,970	36,881,615	21.95	21.53
cuboid object	5	27,390,747	21,052,329	12.15	12.29

Table S3. Per-class IoU of the proposed model for semantic segmentation on the RealSense and Kinect test sets.

Category	RealSense			Kinect		
	Seen	Similar	Novel	Seen	Similar	Novel
pump bottle	37.04	22.50	10.55	52.94	12.10	0.44
tube	56.00	24.81	24.26	64.47	25.54	23.79
container	89.10	68.63	7.06	89.51	66.82	4.68
irregular-shaped object	78.60	52.82	39.76	76.97	35.06	42.14
spherical object	88.51	77.05	0.27	90.65	86.34	2.24
elongated/cylindrical object	70.06	26.52	1.48	71.89	24.27	0.67
cuboid object	95.86	7.81	0.07	96.52	9.24	0.05

Table S4. Ablation study of the MoE configuration for grasp pose estimation performance on the seen, similar, and novel test sets of the RealSense data. Values are reported as AP. The configuration adopted in this study is shaded in gray.

Component	Setting	Seen	Similar	Novel
Reference configuration	8 experts, Top-2, Load-balancing loss, Blocks 3 and 4	66.34	57.21	24.04
Top-K routing	1	67.28	57.53	23.69
	4	67.03	56.47	24.18
No. of experts	4	67.68	57.58	23.31
	16	66.63	58.12	23.88
No. and position of MoE blocks	2 (Blocks 2 and 3)	66.61	58.11	23.47
	1 (Block 4)	67.62	57.65	23.73
Load-balancing loss	No	67.04	56.92	23.73

Table S5. Ablation study of the MoE configuration for semantic segmentation performance on the seen, similar, and novel test sets of the RealSense data. Values are reported as mIoU. The configuration adopted in this study is shaded in gray.

Component	Setting	Seen	Similar	Novel
Reference configuration	8 experts, Top-2, Load-balancing loss, Blocks 3 and 4	73.59	40.02	11.92
Top-K routing	1	74.98	41.80	11.90
	4	73.11	40.37	12.72
No. of experts	4	73.13	40.24	11.67
	16	74.26	42.19	12.49
No. and position of MoE blocks	2 (Blocks 2 and 3)	74.83	42.92	11.99
	1 (Block 4)	73.84	41.77	12.22
Load-balancing loss	No	74.21	40.67	11.11
